# Supplementary material for: Cystitis-Related Bladder Pain Involves ATP-Dependent HMGB1 Release from Macrophages and Its Downstream H2S/Cav3.2 Signaling in Mice
Source: Cells. 2020 Jul 22;9(8):1748. doi: 10.3390/cells9081748 (PMC7463894; doi:10.3390/cells9081748)
Supplement: Supplementary file 1 [file cells-09-01748-s001.pdf]

Supplementary Material

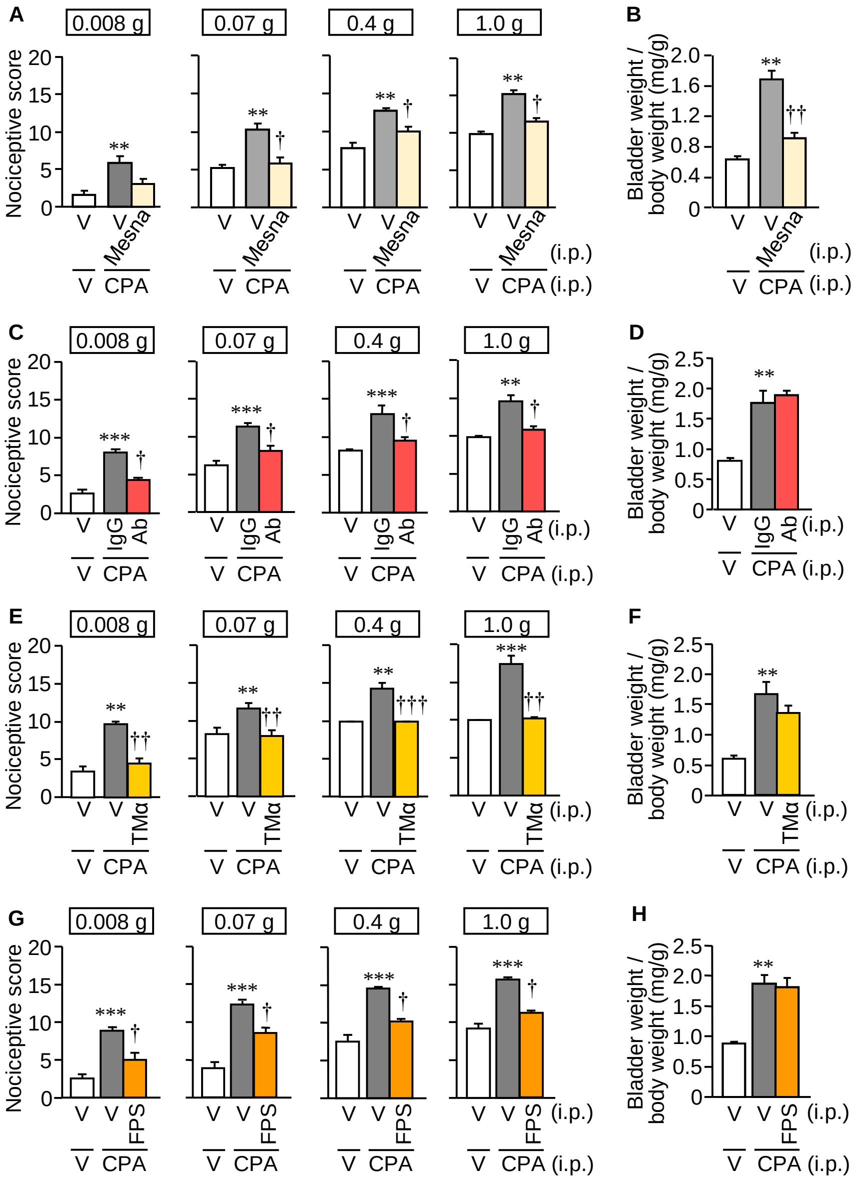

Figure S1. Effect of acrolein quenching with mesna, HMGB1 inactivation with an anti-HMGB1-neutralizing antibody or thrombomodulin alfa, and RAGE blockade with FPS-ZM1 on the CPA-induced referred hyperalgesia (A, C, E, G) and bladder swelling (B, D, F, H) in ddY mice. Mesna was administered orally twice, at 80 mg/kg, 30 min before, and 160 mg/kg, 2 h after i.p. CPA at 400 mg/kg (i.e. a total dose of 240 mg/kg) (A, B), and an anti-HMGB1 antibody (Ab) at 1 mg/kg (C, D), thrombomodulin alfa (TMα) at 10 mg/kg (E, F) or FPS-ZM1 (FPS) at 1 mg/kg (G, H) were administered i.p. 30 min before i.p. CPA at 40. The control mice received i.p. administration of vehicle (V) (A, B, E-H) or a non-immune control IgG at 1 mg/kg (C, D). Referred hyperalgesia was evaluated immediately following observations of nociceptive behavior, i.e. 4 h after i.p. CPA at 400 mg/kg. V, vehicle. Data show the mean with S.E.M. for 5-6 (A-F) or 6 (G, H) mice. *P<0.05, **P<0.01, ***P<0.001 vs. V + V; †P<0.05, ††P<0.01, †††P<0.001 vs. IgG + CPA or V + CPA.

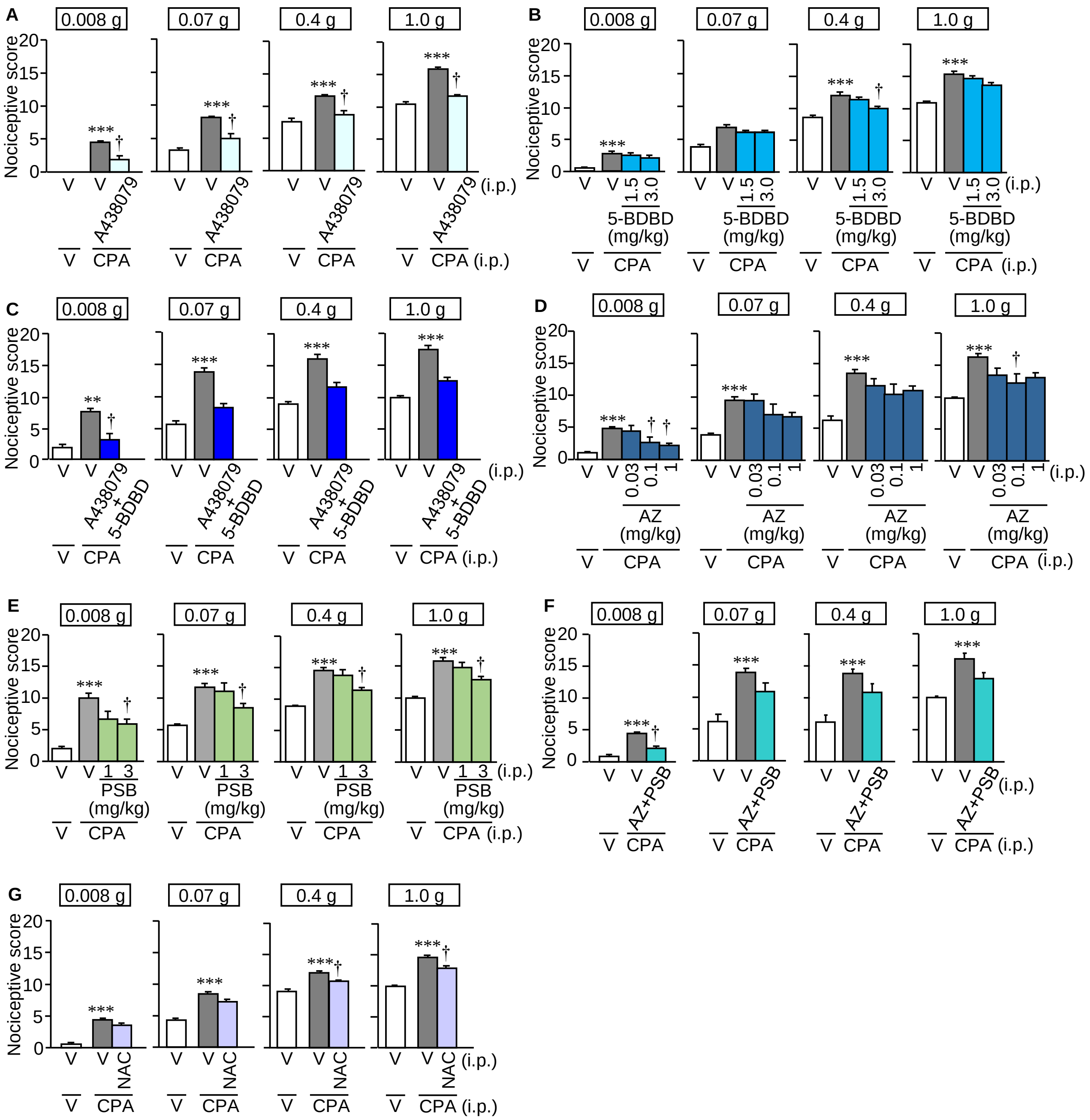

Figure S2. Involvement of purinergic receptors, P2X7 and P2X4, or ROS generation in CPA-induced referred hyperalgesia in ddY mice. The mice received i.p. administration of A438079, a $P2X_7$ antagonist, at 17 mg/kg (A), 5-BDBD, a $P2X_4$ antagonist, at 1.5 or 3 mg/kg (B), A438079 at 17 mg/kg in combination with 5-BDBD at 3 mg/kg (C), AZ10606120 (AZ), a $P2X_7$ antagonist, at 0.03, 0.1 or 1 mg/kg (D), PSB-12062 (PSB), a $P2X_4$ antagonist, at 1 or 3 mg/kg (E), AZ at 1 mg/kg in combination with PSB at 3 mg/kg (F) or N-acetylcysteine (NAC), an antioxidant, at 100 mg/kg (G), 30 min before i.p. CPA at 400 mg/kg. V, vehicle. Data show the mean with S.E.M. for 5-6 (A), 5 (B), 7 (C), 5-10 (D), 5-7 (E), 5-6 (F) or 10 (G) mice. **P<0.01, ***P<0.001 vs. V + V; †P<0.05 vs. V + CPA.

Supplementary Material

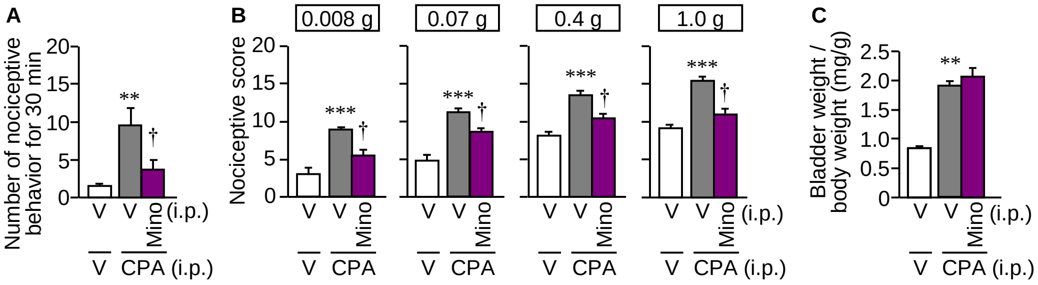

Figure S3. Effect of minocycline on CPA-induced bladder pain-like nociceptive behavior (A), referred hyperalgesia (B) and bladder swelling (C). Minocycline (Mino) at 30 mg/kg was administered i.p. twice, 1 h and 24 h before i.p. CPA at 400 mg/kg. V, vehicle. Data show the mean with S.E.M. for 6-7 mice. **P<0.01, ***P<0.001 vs. V + V. †P<0.05 vs. V + CPA.

Supplementary Material

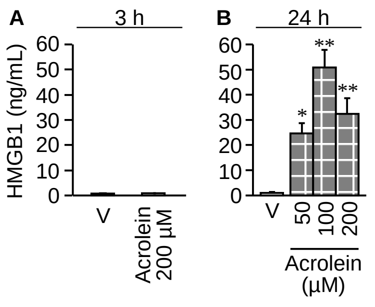

Figure S4. Delayed HMGB1 release from human urothelial T24 cells stimulated with acrolein, a hepatic metabolite of CPA. T24 cells were stimulated with acrolein at 50, 100 or 200 µM for 3 h or 24 h, and the released HMGB1 in the supernatant was determined by ELISA. V, vehicle. Data show the mean with S.E.M for 6 different experiments. $^{*}P<0.05$, $^{**}P<0.01$ vs. V.
